# CCL4 induces inflammatory signalling and barrier disruption in the neurovascular endothelium

**DOI:** 10.1016/j.bbih.2021.100370

**Published:** 2021-10-22

**Authors:** Carolina Estevao, Chantelle E. Bowers, Ding Luo, Mosharraf Sarker, Alexandra Eva Hoeh, Karen Frudd, Patric Turowski, John Greenwood

**Affiliations:** a Institute of Ophthalmology, University College London, 11-43 Bath Street, London, EC1V 9EL, UK; b School of Health and Life Sciences, Teesside University, Stephenson Street, Middlesbrough, TS1 3BA, UK

**Keywords:** Permeability, Chemokines, CCL4, Inflammation, Neuroinflammation, Neurogenesis, BBB, blood-brain barrier, CNS, central nervous system, EC, endothelial cell, F-actin, filamentous actin, hCMEC/D3, immortalized human cerebral microvascular endothelial cell line, MAPK, mitogen-activated protein kinase, SDS-PAGE, Sodium dodecyl sulphate-polyacrylamide gel electrophoresis, VE-cadherin, vascular endothelial cadherin, ZO-1, zonula occludens-1

## Abstract

**Background:**

During neuroinflammation many chemokines alter the function of the blood-brain barrier (BBB) that regulates the entry of macromolecules and immune cells into the brain. As the milieu of the brain is altered, biochemical and structural changes contribute to the pathogenesis of neuroinflammation and may impact on neurogenesis. The chemokine CCL4, previously known as MIP-1β, is upregulated in a wide variety of central nervous system disorders, including multiple sclerosis, where it is thought to play a key role in the neuroinflammatory process. However, the effect of CCL4 on BBB endothelial cells (ECs) is unknown.

**Materials and methods:**

Expression and distribution of CCR5, phosphorylated p38, F-actin, zonula occludens-1 (ZO-1) and vascular endothelial cadherin (VE-cadherin) were analysed in the human BBB EC line hCMEC/D3 by Western blot and/or immunofluorescence in the presence and absence of CCL4. Barrier modulation in response to CCL4 using hCMEC/D3 monolayers was assessed by measuring molecular flux of 70 ​kDa RITC-dextran and transendothelial lymphocyte migration. Permeability changes in response to CCL4 *in vivo* were measured by an occlusion technique in pial microvessels of Wistar rats and by fluorescein angiography in mouse retinae.

**Results:**

CCR5, the receptor for CCL4, was expressed in hCMEC/D3 cells. CCL4 stimulation led to phosphorylation of p38 and the formation of actin stress fibres, both indicative of intracellular chemokine signalling. The distribution of junctional proteins was also altered in response to CCL4: junctional ZO-1 was reduced by *circa* 60% within 60 ​min. In addition, surface VE-cadherin was redistributed through internalisation. Consistent with these changes, CCL4 induced hyperpermeability *in vitro* and *in vivo* and increased transmigration of lymphocytes across monolayers of hCMEC/D3 cells*.*

**Conclusion:**

These results show that CCL4 can modify BBB function and may contribute to disease pathogenesis.

## Highlights

• The chemokine CCL4 induced phosphorylation of P38 in an *in vitro* model of the blood-brain barrier (BBB).
• CCL4 treatment resulted in reduction of plasma membrane VE-cadherin and junctional ZO-1.
• CCL4 induced neurovascular barrier breakdown *in vitro* and *in vivo.*

## 1 Introduction

Neuroinflammation is the inflammatory response to injury or infection in the brain or spinal cord (Fuster-Matanzo et al., 2013) and can lead to either collateral damage or resolution and repair depending on disease setting. In the context of repair, the relationship between neuroinflammation and neurogenesis, the process through which new neurons are generated in the brain, is tied in with different inflammatory components of the immune system, including chemokines, cytokines, and immune cells.

Chemokines, expressed by an array of immune and non-immune cells, including endothelial cells (ECs), have been shown to have a leading role in neuroinflammation and neurodegeneration (Thuc et al., 2015; Ramesh et al., 2013). Engagement of chemokines with their G-protein coupled receptors results in a change in cell behaviour, including migration, proliferation, and activation of inflammatory responses. During inflammation, leukocyte engagement and recruitment across the blood-brain barrier (BBB) is a tightly regulated process involving multiple steps (Springer, 1994). Chemokines play an integral part in this regulation through sequestrating to the endothelial cell glycocalyx where they are closely involved in activating leukocyte adhesion and promoting subsequent chemotaxis and migration to the site of injury (Speyer and Ward, 2011). The functional outcome of chemokines on ECs, however, remains mostly unknown with only a few studies demonstrating chemokine-enhanced BBB permeability effects (Speyer and Ward, 2011). A principal function of the BBB is to maintain tight control over the passage of molecules and cells to and from the central nervous system (CNS) but under many disease conditions, including neuroinflammation, the endothelial barrier becomes hyperpermeable. Accordingly, alterations to properties of the BBB, where barrier function is compromised, have been found in various neurological conditions such as stroke, trauma and neurodegenerative diseases (Barzo et al., 1997; Kortekaas et al., 2005; Zlokovic, 2005; Bell et al., 2010; Iadecola, 2010). Additionally, some of these conditions are also associated with impairments in hippocampal neurogenesis, such as Alzheimer's disease (AD), Parkinson's disease (PD), multiple sclerosis (MS) (Giannakopoulou et al., 2017), stroke and other pathologies with associated neurological and cognitive decline (Wang and Jin, 2015).

Chemokines regulate the recruitment and activation of circulating and resident immune cells in all tissues, including the brain, and orchestrate immune activity during acute and chronic neuroinflammation. The effect of some CCL chemokines on ECs has been explored, but the contributions of the CC chemokine CCL4 (previously known as MIP-1β) in particular, on BBB function, remains poorly understood. CCL4 overexpression in the brain has been reported in a number of diseases including MS (Subileau et al., 2009), AD (Zhu et al., 2014) and PD (Calvani, 2020), and as such may impact on disease progression by causing, amongst other effects, BBB dysfunction. A review on chemokines in MS has highlighted that CCL4 appears to have a role in the pathogenesis of the disease (Ghafouri-Fard et al., 2021). However, it is unknown how it is mechanistically involved, and in particular how it affects the BBB.

The sparse current evidence has shown that CCL4 is able to induce BBB disruption in an animal model of pneumococcal meningitis (Geeta et al., 2013) and has also been shown to increase T cell adhesion to cerebral endothelial cells (Quandt and Dorovini-Zis, 2004). Following ICAM-1 crosslinking, the expression of CCL4 was also found to be upregulated in hCMEC/D3 (Dragoni et al., 2017), suggesting that this chemokine can potentially be induced and act in an autocrine manner on brain endothelial cell function during inflammation. CCL2, from the same subfamily as CCL4, has also been strongly implicated in CNS inflammation and has been shown to increase permeability through disruption of adherens junctions (Dimitrijevic et al., 2006; Roberts et al., 2012). In addition, CCL4 and CCL5 have been shown to enhance adhesion of CD4^+^ T cells to activated brain ECs (Quandt and Dorovini-Zis, 2004). This evidence suggests that chemokine signalling in BBB endothelium, specifically CCL4, could be involved in downstream effects on permeability to macromolecules and immune cells and ultimately to the onset and progression of neuroinflammatory conditions which, in turn, may have direct consequences to neurogenesis.

In this study we determine in a human *in vitro* model of the BBB whether CCL4 can act directly on brain endothelial cell function. We show that CCL4 can alter BBB junctional structure and function placing CCL4 as a potentially important contributing factor to the pathogenesis of this structure in a wide range of neurological conditions.

## 2 Materials and Methods

### 2.1 Human brain microvascular endothelial cells (hCMEC/D3)

HCMEC/D3 (passage 26 and 35) (Weksler et al., 2005) were cultured in EGM™-2 Basal Medium supplemented with FBS, Penicillin Streptomycin (P/S) (1%), hydrocortisone (1 ​mg/mL), ascorbic acid (2.5 ​mg/mL), lipid concentrate, HEPES and bFGF (10 ​μg/mL). Cells were seeded at 8x10^4^ to 1x10^5^ ​cells/mL in collagen I (1:60 in DPBS) coated cell culture plates/flasks and media was changed every other day following a DPBS wash. Cultures were grown to 75–90% confluence before passaging and were used between passage 26 and 35. Culture medium was changed every other day until cells reached 75–90% confluence for renewed passaging. This cell line has been extensively used as a model of the BBB in the context of neuroinflammation (Lopez-Ramirez et al., 2013).

### 2.2 Human umbilical vein endothelial cells (HUVEC)

HUVEC were cultured in EGM-2 medium supplemented with EGM-2 B BulletKit™ (VEGF, IGF, bFGF, hydrocortisone, ascorbate, gentamycin and 2.5% foetal bovine serum (FBS)) as indicated by the manufacturer. cells were seeded at 8x10^4^ - 1x10^5^ ​cells/mL in gelatine-coated cell culture plates or flasks and media was changed every other day. Cells were grown in a humidified atmosphere at 37 ​°C in 5% carbon dioxide (CO2) and up to 75–90% confluence and used between passage 2 and 5.

### 2.3 Immunoblotting

Cells were lysed in lysis buffer (4% SDS, 10% 2-mecaptoethanol, 20% glycerol, 0.004% bromophenol blue 0.125 ​M Tris HCl). Samples were collected and denatured at 100 ​°C for 5 ​min and centrifuged and frozen at −20 ​°C until further use. Alternatively, cells were lysed in RIPA buffer with protease and phosphatase inhibitors (to avoid proteolysis and preserve phosphorylation of proteins), and centrifuged. Supernatants were further denatured using lysis buffer. Sodium dodecyl sulphate-polyacrylamide gel electrophoresis (SDS-PAGE) and immunoblotting were performed as previously described (Turowski et al., 1999) using antibodies against the proteins of interest: CCR5 (Abcam) was used at 1:3,000, HSC70 (Sigma) at 1:10,000 and P–P38 MAPK (T180/Y182) at 1:3000 (Abcam), anti-Human VE-Cadherin Monoclonal Antibody (R&D Systems) at 1:3000 in 0.1% PBSA. Secondary antibodies were used at 1:10,000 in 0.1% PBSA. For quantitative analyses immunoblots were scanned and quantified using the NIH imaging software ImageJ.

### 2.4 Immunofluorescence

HCMEC/D3 were grown on collagen I coated 35 ​mm^2^ Petri dishes and stimulated with CCL4 for 5, 10, or 60 ​min, fixed in 4% paraformaldehyde and blocked in PBSA (0.5% with sodium azide). Nuclei of cells were visualised by the addition of 1 ​μg/mL Hoechst 33258 (bis-benzminide) or DAPI in the secondary antibody staining solution. The dishes were stored at 4 ​°C, mounted, and imaged using the Zeiss LSM 710 confocal microscope. Optionally, cells were permeabilised using ice cold acetone at −20 ​°C. Cells were then incubated with primary and secondary antibody at 37 ​°C in a humidified chamber. To reveal surface VE-cadherin, permeabilisation was performed after the first primary antibody incubation. Anti-Human VE-Cadherin Monoclonal Antibody (R&D Systems) and VE-cadherin Antibody (C-19) (Santa Cruz Biotechnology) were used at 1:200, P–P38 MAPK (T180/Y182) (Cell Signalling Technology) was used at 1:3000 and ZO-1 (Thermofisher) was used at 1:50, in 0.1% PBSA. Secondary antibodies were used at 1:300 in 0.1% PBSA.

### 2.5 Permeability measurement in hCMEC/D3

hCMEC/D3 cells were grown on 12-mm transwell filters with 0.4 ​μm pore size. The technique was performed as described previously (Martins et al., 2013). 70 ​kDa Rhodamine B isothiocyanate (RITC)-dextran was added at 1 ​mg/mL to the apical side of the transwell filters. 50 ​μl samples were collected from the basal chamber (and replaced with EBM-2 culture media) at 15 ​min intervals for 180 ​min with our without the addition of CCL4. Fluorescence of samples was measured in a Tecan Safire Fluorescence Reader, plotted against time and permeability changes were determined from linear slope changes. Statistical analysis was done using a two tailed Student's t-test. Analysis performed using GraphPad Prism version 5.0 for Windows (GraphPad Software, San Diego CA USA).

Recombinant Human MIP-1β (CCL4) (used in all experiments) was purchased from Peprotech (cat. Number 300-09). As per manufacturer's indication, purity is ​≥ ​98% by SDS-PAGE gel and HPLC analyses (Recombinant HumanP-1β, 2021).

### 2.6 Primary T cells

Human CD4^+^ cells were isolated from peripheral blood from healthy volunteers using Ficoll® Plaque Premium and positive selection on CD4^+^ MACS beads (Miltenyi) as previously described (Martinelli et al., 2008). CD4^+^T cells were resuspended in RPMI-1640 supplemented with 10% FCS, 100 U/ml penicillin, 100 ​μg/ml streptomycin,1 ​mM sodium pyruvate, 1 ​mM nonessential amino acids, 2 ​mM l-Glu, and 50 ​μm β-mercaptoethanol supplemented with 25 ​ng/mL of IL-2 at a density of 500,000 ​cells/mL.

### 2.7 T-lymphocyte endothelial transmigration

Technique performed as previously described (Adamson, Etienne, Couraud, Calder, Greenwood). CCL4 was added to hCMEC/D3 for 30min, washed off, and T cells resuspended in HBSS were added to the wells. Lymphocytes were left to adhere and migrate for 30 ​min in an incubator and T lymphocytes that did not adhere were gently washed off using HBSS at 37 ​°C. For imaging purposes, plates were mounted on a phase contrast microscope (Zeiss 200 ​M). Mean migration rates and standard mean errors were calculated from at least three independent experiments. Statistical analysis was carried out using a two-tailed Student's t-test. Analysis performed using GraphPad Prism version 5.0 for Windows (GraphPad Software, San Diego CA USA).

### 2.8 *In vivo* permeability measurements in pial microvessels

The method used in this study, and its theoretical basis, have been described previously (Easton and Fraser, 1994). The experiments were performed on male Wistar rats (25–30 days) within guidelines set by The Animals (Scientific Procedures) Act 1986 and the EU Directive 2010/63/EU guidelines for animal experiments.

The rats were anesthetised, the dura and arachnoid were removed, thus exposing the microcirculation of the surface of the brain and leaving it free of any diffusion barrier towards the superfusing solution. Sulforhodamine B was added to the brain microcirculation into the carotid artery and viewed under 540/25 ​nm illumination. The signal was captured through a microscope coupled to an image-intensifier camera (Hamamatsu). Video microscopy (1 frame per 2s) recorded the time-dependent loss of dye in a single vessel, occluded by a glass probe. A baseline recording was established for 20–60 ​s. To obtain measurements, the flow of superfusing solution was stopped so that a pool was formed between the brain and the microscope's water immersion lens. Dye was injected into the circulation and the probe was placed over the vessel to be analysed. Remaining dye in the circulation was removed by the blood flow. For the abluminal CCL4 additions, the compounds were applied to the pool on the brain surface. Image analysis and densitometry was performed using ImageJ 1.45s (NIH). At the end of the experiment animals were culled by an overdose of the anaesthetic.

The mean pixel intensity was measured for each vessel tested for each frame/2 ​s and plotted against time. Data was normalized to diameter of the vessel according to pre-set graticule that correlates vessel diameter in a 20x Olympus field (used for the experiment) with 10 ​μm grid. Mean pixel intensity before and after addition and corresponding SEM were calculated and a paired *t*-test was used to compare differences between the two time sets (before and after additions).

### 2.9 Intravitreal injections and fundus fluorescein angiography

Experiments performed on adult C57BL/6J mice within guidelines set by The Animals (Scientific Procedures) Act 1986 C57BL/6J and the EU Directive 2010/63/EU guidelines for animal experiments.

Adult C57BL/6J animals were anaesthetised using an intra-peritoneal injection of Ketamine (60 ​mg/kg) and Domitor® (10 ​mg/kg). For intravitreal injections, 1 ​μL of CCL4 (at 1 ​mg/mL diluted in 0.1% PBSA) to give a final dose of 1 ​μg per eye. Control animals received 1 ​μL of vehicle. FFA was carried out 10 ​min following CCL4 injections using a Micron III: pupils were dilated with 1% tropicamide, eyes kept moist with Viscotears®, and light fundus images acquired followed by green fluorescence images at 1.5 and 7 ​min following subcutaneous injection of 2% fluorescein. Statistical analysis carried out using Student's *t*-test for variance of mean values.

## 3 Results

### 3.1 CCR5 expression in hCMEC/D3

**Fig. 1:**
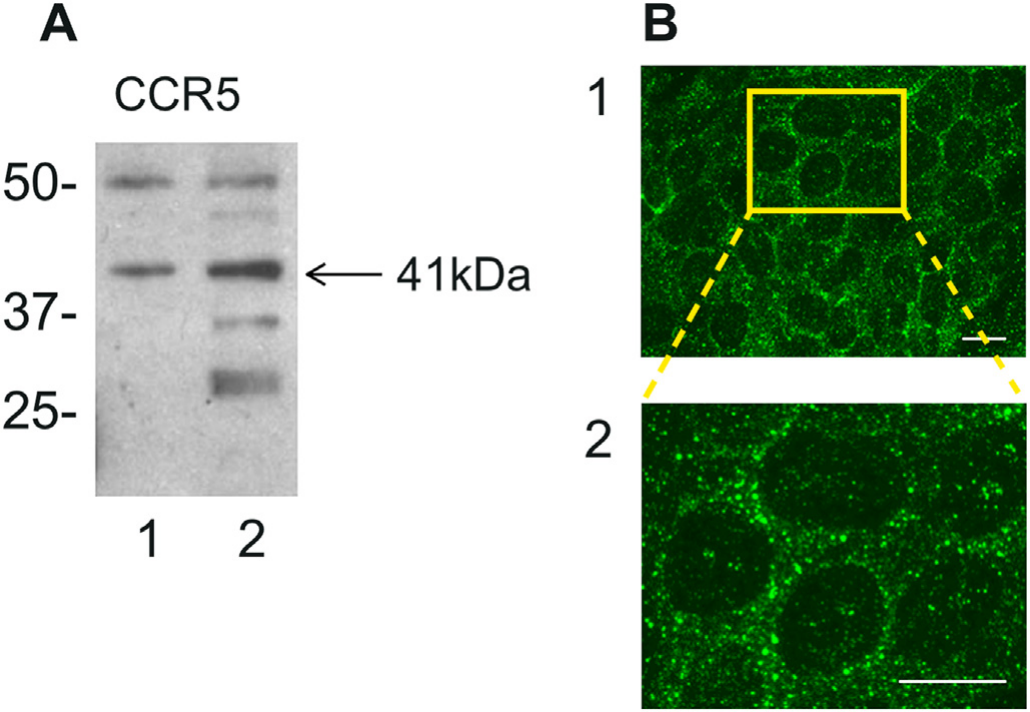
**CCR5 expression in hCMEC/D3**. A- Western blot of endothelial cell lysates from HUVEC (lane 1) and hCMEC/D3 (lane 2). The position of CCR5 predicted by its molecular weight is arrowed. B – 1) CCR5 expression in hCMEC/D3 detected by immunofluorescence showing punctate cytoplasmic membrane staining concentrated in the periphery of each cell; Yellow box highlights regions of images magnified in the Zoom panel 2) Zoom detail. Scale bar: 10 ​μm. (For interpretation of the references to colour in this figure legend, the reader is referred to the Web version of this article.)

The expression of CCR5, the main receptor for CCL4 (Rottman et al., 1997), was examined in the brain microvascular endothelial cell line hCMEC/D3 and HUVEC using specific antibodies. Immunoblots of whole cell hCMEC/D3 lysates revealed a protein band, which co-migrated with a band in HUVEC lysates and at the predicted molecular mass of 40.6 ​kDa for CCR5 (Fig. 1A). Furthermore, when used in indirect immunofluorescence the CCR5 antibody revealed strong staining in hCMEC/D3, which was concentrated in the cell periphery (Fig. 1B).

### 3.2 CCL4 treatment mediates p38 activation

**Fig. 2:**
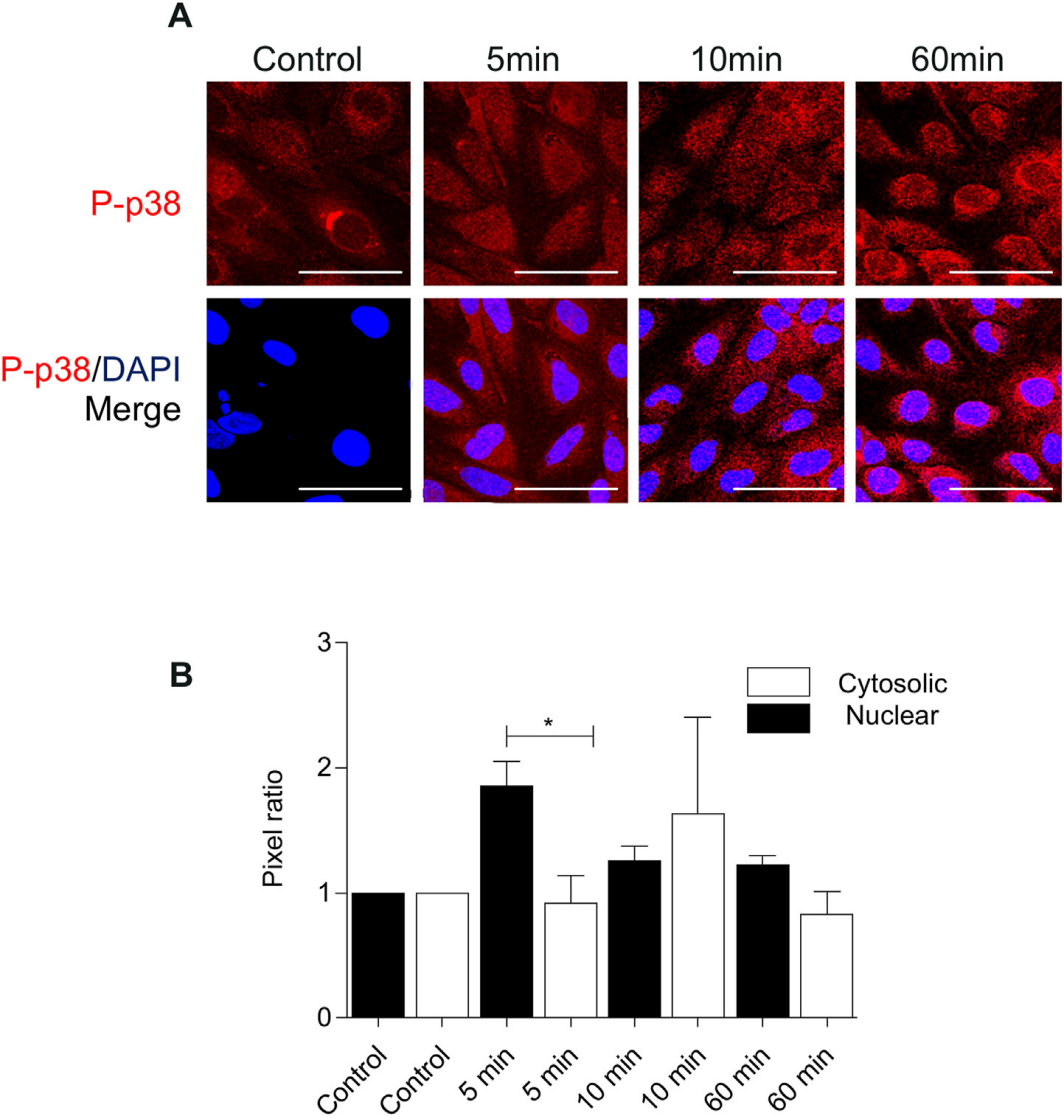
**- CCL4 treatments mediated p38 activation.** Distribution of P–P38 in hCMEC/D3 following CCL4 treatment (100 ​ng/mL). Cells were stained for phospo-p38 and nuclear DNA. A- Images show full projection of confocal stacks through the full thickness of the cells. Scale bar 50 ​μm. Images were taken on Zeiss LSM700. B - Cytosolic/nuclear phospho-protein quantification, normalized to control. Results are expressed as average means ​± ​SEM. Statistical analysis performed using One-way ANOVA and a Dunnet post hoc test for statistical analysis of mean value variances. ∗, P ​< ​0.05, P ​= ​0.0002, F ​= ​11.24 and dF ​= ​19.

P38 mitogen-activated kinase is a key regulator of neurovascular barrier function (Dragoni et al., 2017; Hudson et al., 2014). Stimulation of hCMEC/D3 with CCL4 induced transient phosphorylation of p38 with a rapid induction at 5- and 10-min post CCL4 treatment and a return to control levels at 60 ​min (Fig. 2A). Accumulation of phospho-p38 was observed both in cytoplasm and nucleus, but notably significantly more marked in the nucleus, especially at 5min post treatment, demonstrating that CCL4 induced activation of this key signalling molecule at the BBB (Fig. 2B).

### 3.3 Stress fibre formation in response to CCL4 treatment

In most cells, including ECs, p38 activation results in the induction of stress fibre formation (Huot et al., 1997). Stress fibres are part of the actin cytoskeleton on ECs and allow for morphological change to occur in response to stimuli (Schnittler et al., 2014). Stress fibres, specifically, cytoplasm transversing actin fibres form in the endothelium when it becomes activated in inflammatory conditions, wound healing and other situations where homeostasis is compromised (Schnittler et al., 2014; Abu Taha, Schnittler).

**Fig. 3:**
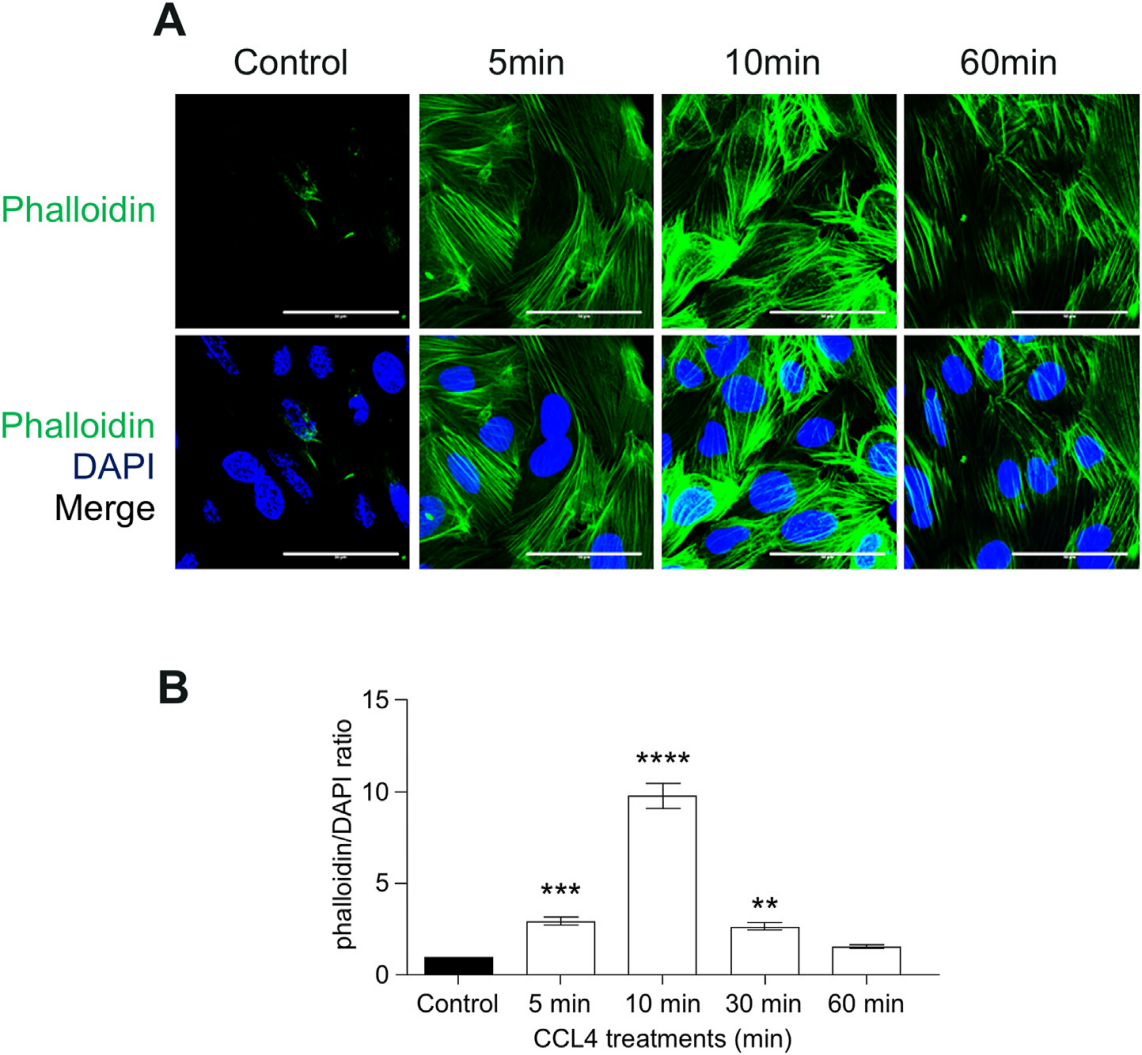
**– Stress fibre formation in response to CCL4 treatment.** hCMEC/D3 were left untreated or treated with CCL4 (100 ​ng/mL) for 5, 10, 30 and 60min, fixed and immunostained for phalloidin and nuclear DNA. A- Images show full projection of confocal stacks through the full thickness of the cells. Images were taken on Zeiss LSM700. Scale bar 50 ​μm. B - Fibre intensity mean area average ​± ​SEM of phalloidin area/DAPI, normalized to control. Statistical analysis used One-way ANOVA and Dunnet post hoc test. ∗∗, 0.001<P ​< ​0.01; ∗∗∗, P ​< ​0.001; ∗∗∗∗, P ​< ​0.0001, P ​= ​0.0039, F ​= ​114.3, and dF ​= ​19.

Therefore, we next assessed whether CCL4 induced stress fibre formation. Strong stress fibres were observed in hCMEC/D3 following treatment with CCL4 (Fig. 3A). The induction time course of F-actin was very similar to that of phospho-p38, with a significant increase from 5min and peaked at 10min with a 10-fold increase in actin intensity (Fig. 3B).

### 3.4 Decrease of junction ZO-1 in response to CCL4

**Fig. 4:**
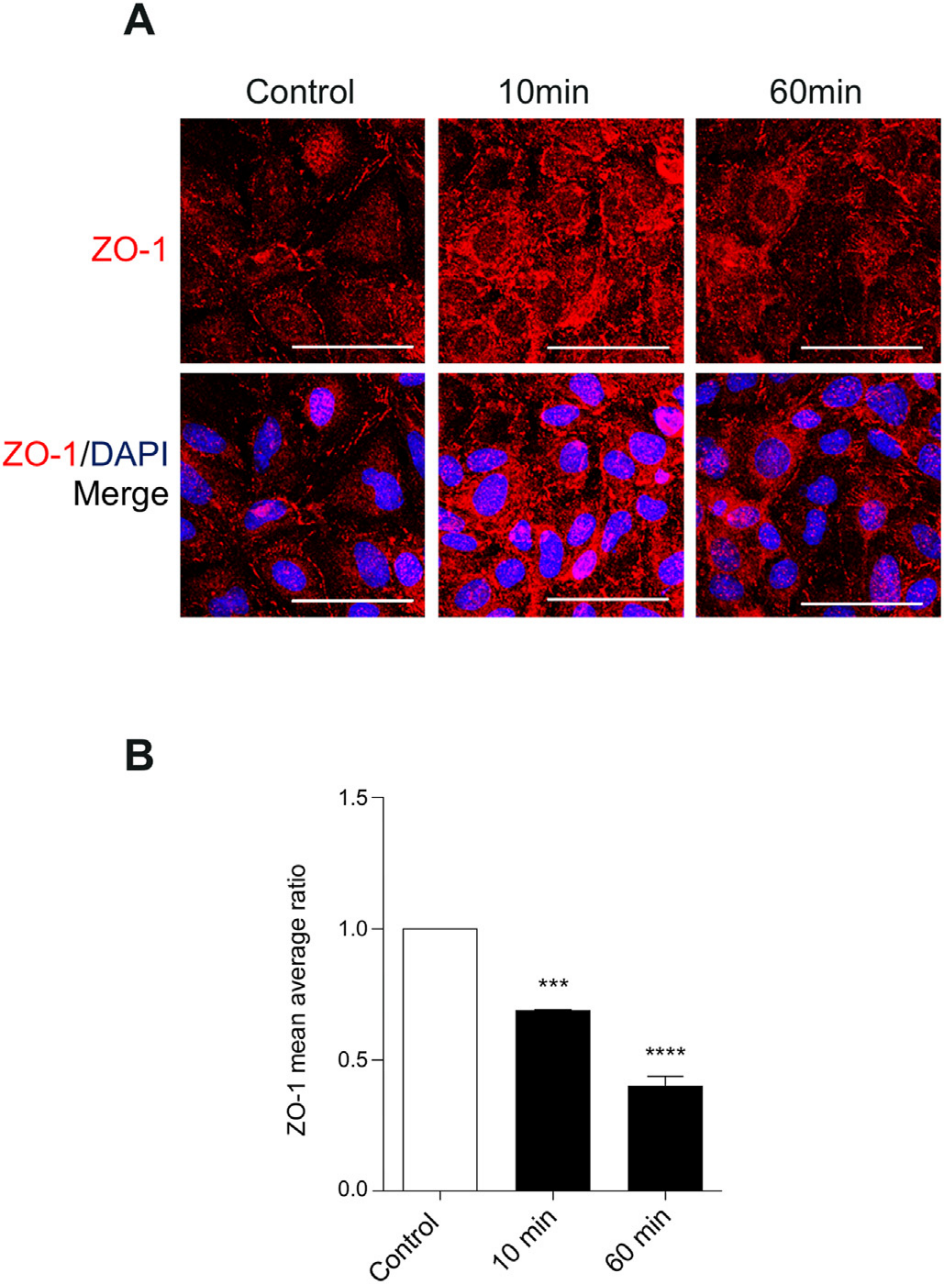
**– Decrease of junction ZO-1 in response to CCL4.** hCMEC/D3 were left untreated or stimulated with CCL4 (100 ​ng/ml) for the indicated times before fixation and staining for ZO-1 and nuclear DNA. A - Images show full projection of confocal stacks through the full thickness of the cells. Images were taken on Zeiss LSM700. Scale bar 50 ​μm. B - Intensity of junctional ZO-1 was determined by average mean area of junctional ZO-1, normalized to nuclear stain. Shown are mean normalized intensities −/+ SEM. Statistical analysis used One-way ANOVA and Dunnet post hoc test. ∗∗∗, P ​< ​0.001, P ​< ​0.0001, F ​= ​198.9, and dF ​= ​8.

To test whether CCL4 treatment altered brain EC junctions, the tight junction associated protein ZO-1 was investigated. Treatment of hCMEC/D3 with CCL4 resulted in a consistent alteration to the cellular distribution of ZO-1, which we observed as early as 10 ​min post treatment (Fig. 4A). Quantitative image analysis revealed significant reductions of ZO-1 at the junctions by *circa* 30% and 60% at 10 and 60 ​min respectively, demonstrating that CCL4 has a profound effect on a key junctional protein (Fig. 4B).

### 3.5 CCL4 significantly reduces VE-Cadherin signal from the plasma membrane

All of the above was compatible with signalling leading to endothelial barrier dysfunction, which is specifically linked to re-distribution of the adherens junction protein VE-cadherin (Orsenigo et al., 2012; Dragoni et al., 2021; Wessel et al., 2014), including the interplay of VE-cadherin and actin filaments that can be seen in junctional remodelling (Schnittler et al., 2014).

**Fig. 5:**
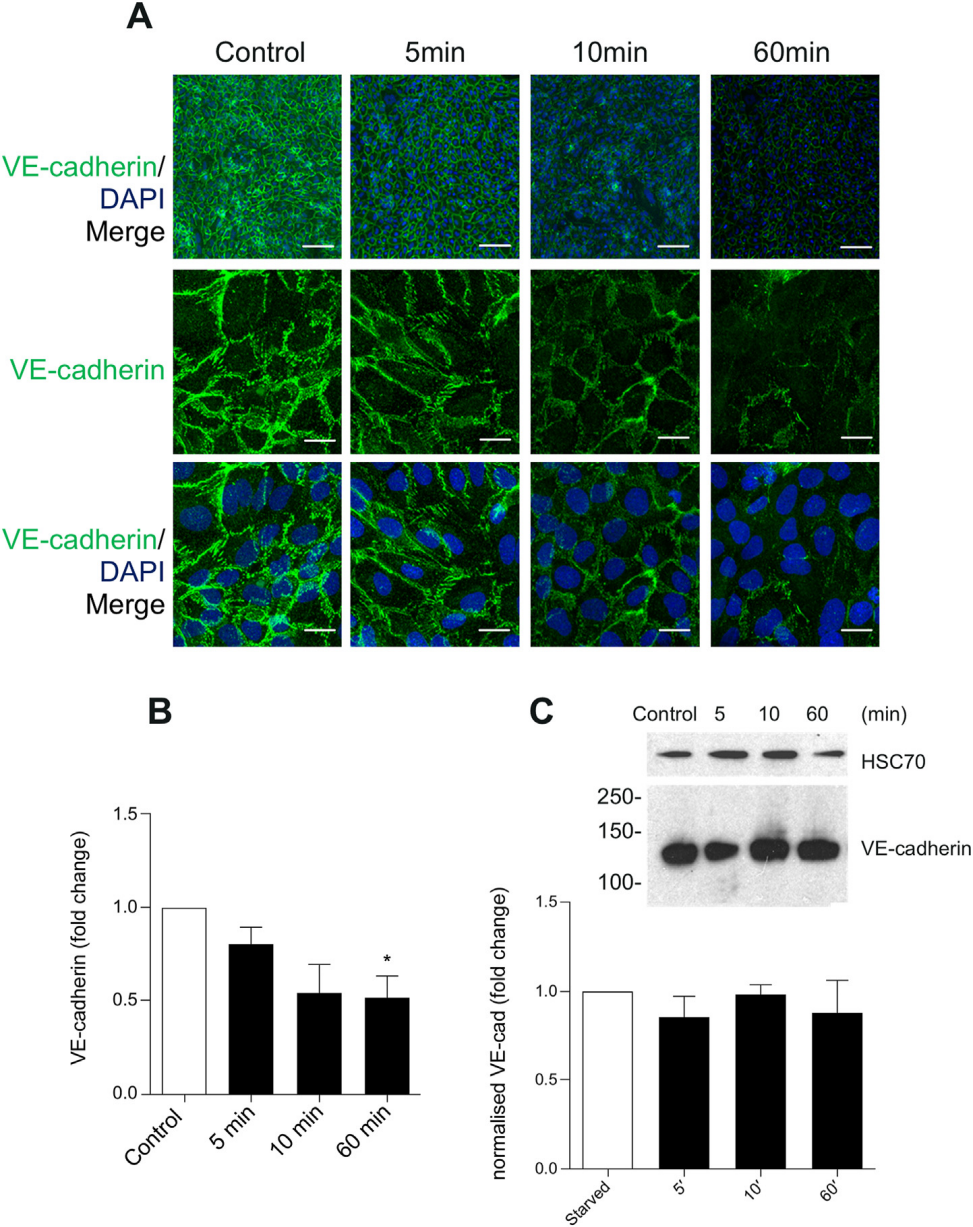
**– Decrease in VE-cadherin in response to CCL4.** hCMEC/D3 were left untreated or treated with CCL4 (100 ​ng/mL) for 5, 10 and 60 ​min, fixed and stained for VE-cadherin and nuclear DNA. A- Images show full projection of confocal stacks through the full thickness of the cells. Images were taken on Zeiss LSM700. Scale bar 50 ​μm (top row) or 10 ​μm (middle and bottom row). B - Mean area average ​± ​SEM of VE-cadherin stain, expressed as VE-cadherin fold change. Statistical analysis used a One-way ANOVA and Dunnet post hoc test. ∗p ​< ​0.05. P ​= ​0.0462, F ​= ​3.992, and dF ​= ​15. C – Western blot and analysis of total VE-Cadherin. Normalized Mean VE-cadherin ​± ​SEM, normalized to HSC70 is shown as fold change. Statistical analysis used a One-way ANOVA and Dunnet post hoc test.

To test whether CCL4 induced changes in VE-cadherin, hCMEC/D3 were treated with CCL4 and VE-cadherin distribution was investigated using several immunofluorescence protocols. Overall, there was a gradual reduction in junctional VE-cadherin, which was significantly reduced by approximately 50% after 60 ​min of CCL4 treatment (Fig. 5A and B). However, there was no significant reduction in response to CCL4, when VE-cadherin levels were assessed in whole cell lysates (Fig. 5C), indicating that the reduced signal detected by immunofluorescent microscopy was due to epitope masking and/or redistribution.

**Fig. 6:**
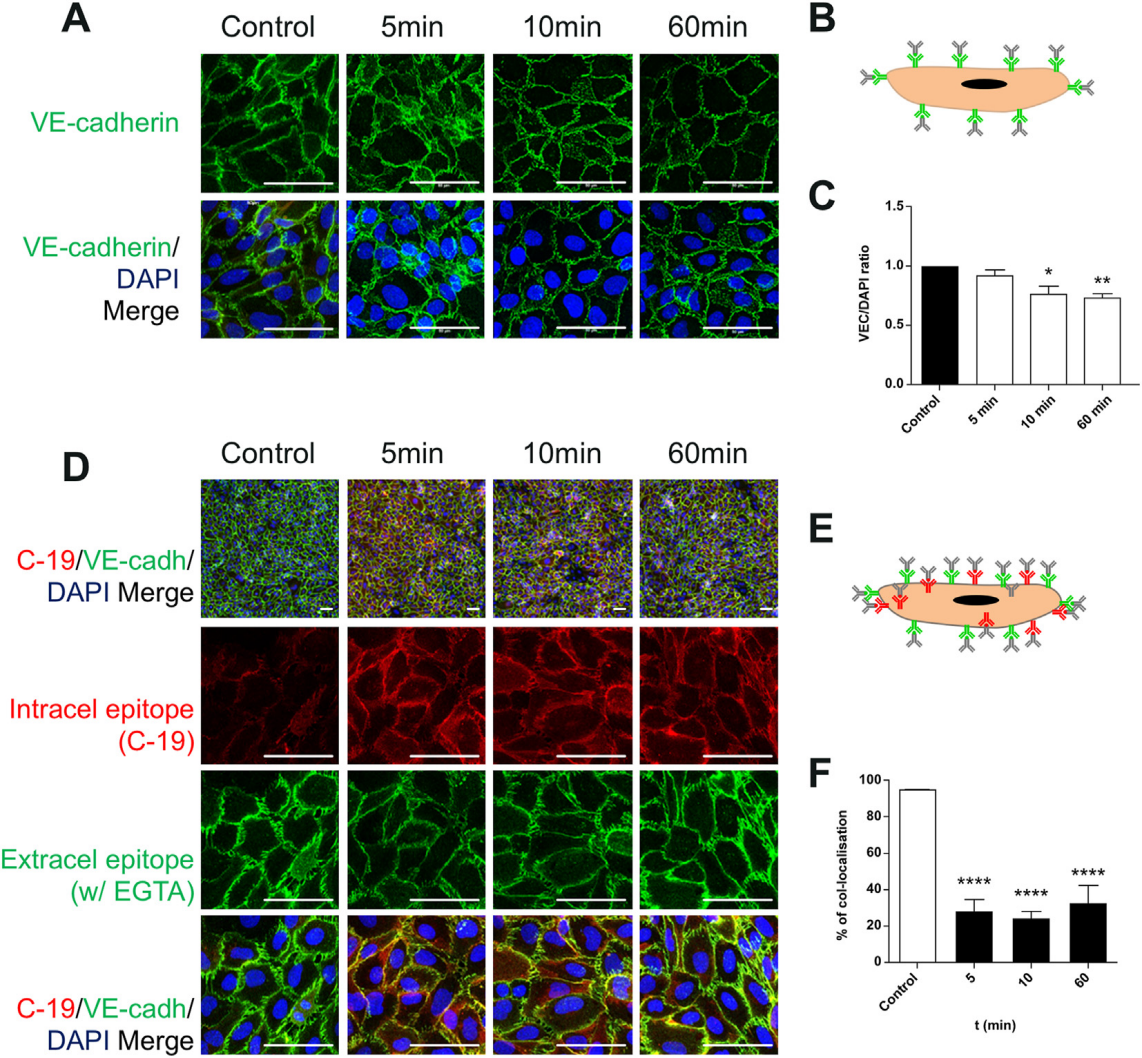
**- CCL4 significantly reduces VE-Cadherin signal from the plasma membrane.** VE-cadherin expression following CCL4 treatments for 5-, 10-, 30- and 60-min. Confluent hCMEC/D3 were left untreated or were treated with CCL4 (100 ​ng/mL) for the above mentioned timepoints, stained for the extracellular epitope of VE-cadherin and nuclear DNA. A - Images show full projection of confocal stacks through the full thickness of the cells. Images were taken on Zeiss LSM700. Scale bar 50 ​μm. B- Experimental detail: single endothelial cell and primary (green) and secondary antibodies (grey). C-– Mean area average ​± ​SEM of VE-cadherin stain, normalized to control. Statistical analysis used One-way ANOVA and Dunnet post hoc test. ∗, P ​≤ ​0.05; ∗∗, P ​≤ ​0.01. P ​= ​0.0095, F ​= ​4.512, and dF ​= ​32. D - VE-cadherin expression following CCL4 treatments for 5, 10, 30 and 60 min hCMEC/D3 were left untreated or were treated with CCL4 (100 ​ng/mL) for the above mentioned timepoints, stained for the extracellular epitope of VE-cadherin, intracellular epitope (C-19) and nuclear DNA. VE-cadherin Z stacks were taken on Zeiss LSM700 using a 63x and a 10x objective. A - Images show full projection of confocal stacks through the full thickness of the cells. Images were taken on Zeiss LSM700. Scale bar 20 ​μm (top row only) or 50 ​μm. E − Experimental detail: single endothelial cell, and primary (green and red) and secondary (grey) antibodies. F - JACoP plugin on image J was used to calculate the coefficient between the red/green channels for pixel co-localis ation in 10x images. Statistical analysis performed using a One-way ANOVA and Dunnet post hoc test. ∗, P ​≤ ​0.05. analysis performed using a One-way ANOVA and Dunnet post hoc test. ∗∗∗, P ​< ​0.001. P ​= ​0.0001, F ​= ​28.19, and dF ​= ​11. (For interpretation of the references to colour in this figure legend, the reader is referred to the Web version of this article.)

To assess the level of cell surface VE-cadherin directly, live hCMEC/D3 cells were incubated in a buffer containing the calcium chelator EGTA to disrupt homotypic VE-cadherin. Subsequently, VE-cadherin was detected by indirect immunofluorescence using an extracellular epitope-specific antibody (see Fig. 6B for illustration). There was a significant reduction in VE-cadherin signal (Fig. 6A), with significans decreases from 10 ​min sustained during the course of CCL4 treatment (Fig. 6C), indicating a loss of VE-cadherin from the junctional cell surface.

Next, internalisation of VE-cadherin was directly assessed by comparison between plasma membrane localised (i.e., accessible using extracellular specific antibodies in non-permeabilised cells) and total VE-cadherin (stained for using a carboxy-terminal antibody following cell permeabilisation)(see Fig. 6E for illustration). In control cells total and plasma membrane VE-cadherin staining largely overlapped (Fig. 6D), indicating that the bulk of the protein was engaged in paracellular cell surface junctions. Significantly, following CCL4 stimulation, staining was detected in the channel for total VE-cadherin, which did not overlap with the signal for surface VE-cadherin (Fig. 6D and F). Indeed, this additional signal was observed as early as 5 ​min following CCL4 treatment and was localised in close proximity to the junctions spreading towards the inside of the cells, a localisation consistent with internalisation (Dragoni et al., 2021).

### 3.6 CCL4 induces neurovascular barrier breakdown *in vitro* and *in vivo*

**Fig. 7:**
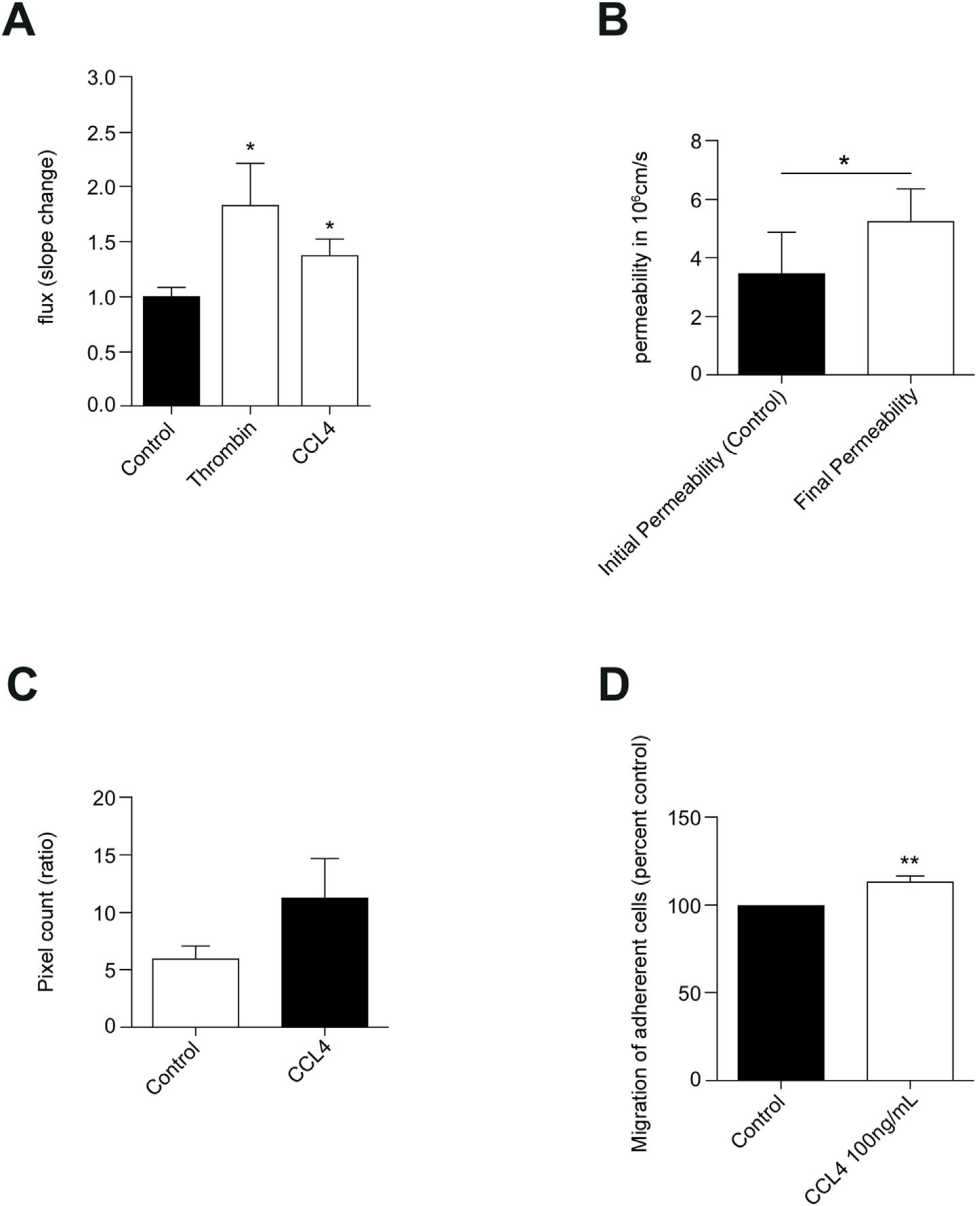
**- CCL4 induces neurovascular barrier breakdown *in vitro* and *in vivo*.** A -Transendothelial flux changes in response to CCL4 in hCMEC/D3. Transendothelial flux was measured in confluent hCMEC/D3 grown in collagen coated transwells over a period of 180 ​min. Response to apical 0.1% PBSA (Control), Thrombin (1U/ml) and CCL4 (100 ​ng/mL). Data represents the mean slope change after addition of treatments. Means ​± ​SEM of four independent experiments. ∗, P ​< ​0.05. B - *In vivo* measurements of permeability in pial microvessels following CCL4 addition. Mean permeability change in response to abluminal administration of CCL4 (50 ​μg/mL). Data are means ​± ​SEM of at least 7 vessels in three adult male Wistar rats. ∗, P ​< ​0.05. Statistical test carried out using paired Student t-test. C - Fluorescein leakage following CCL4 intravitreal injection. Mice injected with CCL4 (10 ​μg/mL, intravitreal injection) 15 ​min prior to imaging. Images obtained at 1.5- and 7-min post fluorescein injection were thresholded and converted to black and white. Pixel number was measured, and a ratio 1.5 ​min/7 ​min was calculated. Shown are means ​± ​SEM of four C57BL/6J adult male mice. Statistical analysis carried out using Student's *t*-test for variance of mean values. Data non-significant. D - Lymphocyte migration across hCMEC/D3 monolayer. CCL4 (100 ng/mL) was incubated with hCMEC/D3, T cells were added and allowed to migrate. Values of migration are expressed as mean percentages of control from at least 4 independent experiments ​± ​SEM. Variances of mean values were statistically analysed by the Student's *t*-test. ∗∗, 0.001<P ​< ​0.01.

Actin stress fibre induction and junction protein redistribution in ECs are commonly associated with altered permeability. Thus, we examined monolayer permeability of hCMEC/D3 cells in response to CCL4. The flux of a 70 ​kDa dextran fluorescent tracer was significantly enhanced in response to CCL4 administration albeit somewhat less than that seen in response to thrombin, a powerful permeability factor of ECs (Fig. 7A). Following these results, the effects of CCL4 *in vivo* were investigated. A similar, significant *circa* 1.5-fold increase in sulforhodamine permeability was also observed in Wistar rat pial vessels in response to CCL4 *in vivo* (Fig. 7B). Also *in vivo*, increased leakage as measured by fluorescein angiography was also observed in the mouse retinal vasculature in response to intra-ocular CCL4 injections (Fig. 7C). However, this did not quite reach significance in 7 independent measurements. BBB barrier breakdown to molecules often coincides with its breakdown to immune cells (Martinelli et al., 2014). Thus, we investigated if CCL4 facilitated T cell migration across a monolayer of ECs. For this hCMEC/D3 cells were pre-treated or not with CCL4 and then co-cultured with CD4^+^ cells isolated from peripheral blood. CCL4 pre-treatment resulted in a modest, but significant, increase in T-lymphocyte transendothelial migration (Fig. 7D).

**Fig. 8:**
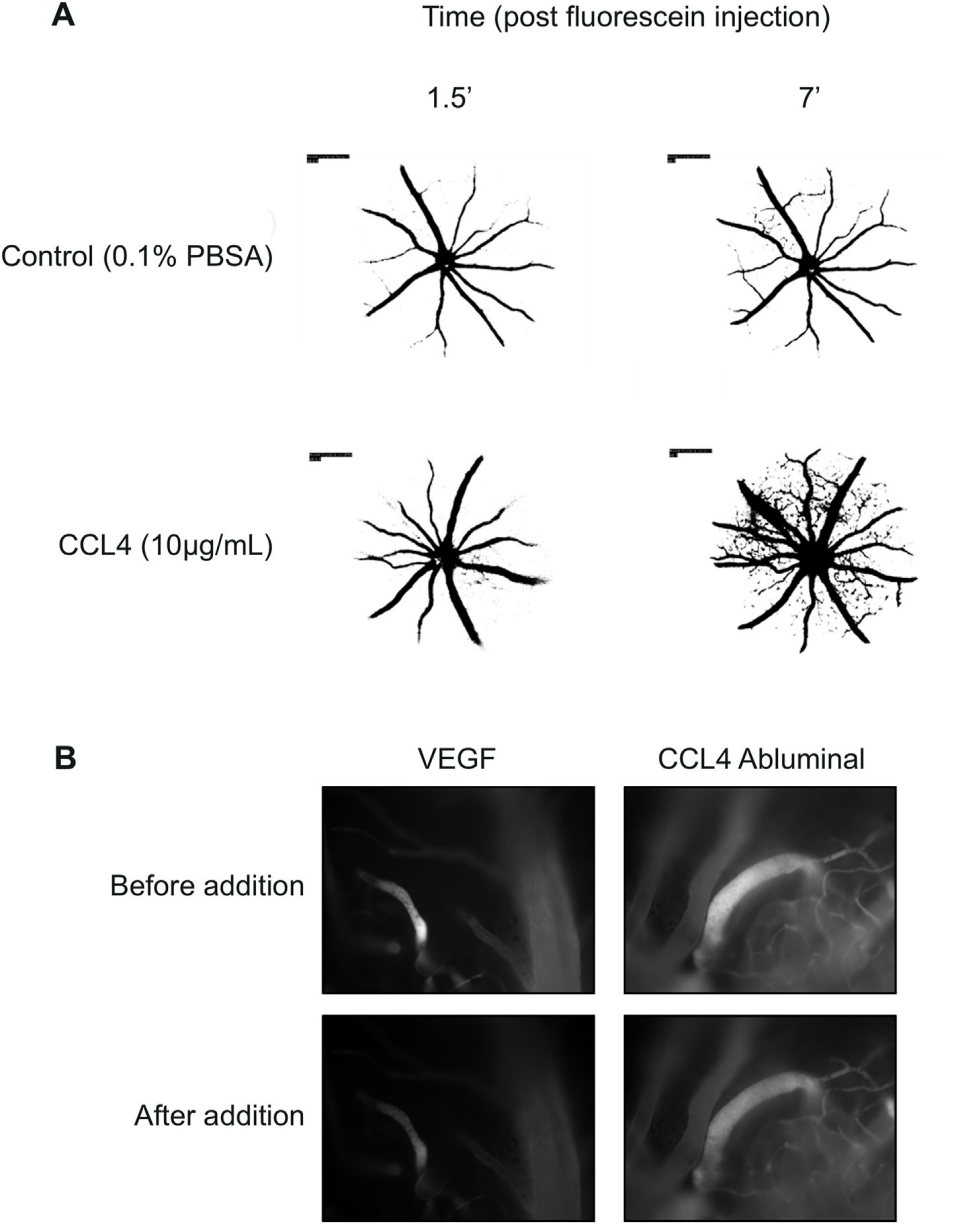
**– Representative images for in vivo experiments.** A- Fluorescein leakage following CCL4 intravitreal injection. A- Representative binary images of the fundus fluorescein angiography of retinas of adult mice injected with CCL4. Mice injected with CCL4 (10 ​μg/mL, IVT injection) 15 ​min prior to imaging and B - In vivo measurements of permeability in pial microvessels following CCL4 abluminal addition at 50 ​μg/mL.

Fig. 8 illustrates the retinal fluorescein angiography of control and CCL4 injected mice retinas (A, representative image) and the pial microvasculature of Wister rats, injected with VEGF (positive control) and CCL4 (B, representative pictures).

## 4 Discussion

CCL4 is considered an important chemokine in many neuroinflammatory conditions. However, whilst other chemokines have demonstrated effects on the integrity of the BBB, the biological response of BBB ECs to CCL4 has not yet been investigated. Here we show that CCL4 induces biochemical and functional changes in BBB ECs, the characteristics of which are consistent with BBB dysfunction.

Expression data for CCR5 in BBB ECs varies; whilst single cell RNAseq analysis of the mouse brain vasculature reveals only very low to no CCR5 RNA expression in BBB ECs (Vanlandewijck et al., 2018), there is clear evidence that BBB ECs express functional CCR5 (Andjelkovic and Pachter, 2000; Edinger et al., 1997), indicating that BBB ECs possess the machinery to respond to CCL4 stimulation. Whilst there are discrepancies in the literature, there are several reports that indicate the human EC cells express CCR5 (Li et al., 2013; Kanmogne et al., 2000; Maguire et al., 2014), in addition to several reports on murine tissues (Vanlandewijck et al., 2018; Andjelkovic and Pachter, 2000; Edinger et al., 1997). The discrepancies in expression can be attributed to different tissues tested and experimental methods used; nevertheless, our report confirms the expression of CCR5 by hCMEC/D3, a cell line derived from human temporal lobe microvessels.

Both stress fibre induction and structural and functional changes to endothelial cell junctions are often linked to p38 activation. Consistent with our observation of actin cytoskeletal reorganisation, p38 is reportedly involved in cytoskeletal destabilisation and changes in endothelial cell permeability (Li et al., 2015; Koss et al., 2006). In addition, tight junction proteins can be modulated by chemokines and mitogen-activated protein kinase (MAPK) signalling and cytoskeletal rearrangement are involved in the process (Hudson et al., 2014; Shahabuddin et al., 2006). Accordingly, p38 has been shown previously to modulate the expression and distribution of tight junction proteins, including ZO-1, linking this signalling pathway into the regulation of vascular permeability. Indeed, MAPK kinases are involved in co-modulating tight junction protein phosphorylation and thus are able to regulate paracellular transport and hence barrier permeability (Gonzalez-Mariscal et al., 2008).

Chemokines can disturb the organization of junctions in endothelial cells (Gonzalez-Mariscal et al., 2008; Wallez and Huber, 2008). For example, in brain endothelial cells recent work has shown that the chemokine CCL2 disrupts tight junctions, including altering ZO-1 distribution, and that this results in increased BBB permeability (Dimitrijevic et al., 2006; Luissint et al., 2012). Similarly CKLF1, a C–C chemokine, has been shown to be involved in downregulation of ZO-1 expression and BBB disruption (Kong et al., 2016). Our study adds CCL4 to this list of junction modulating chemokines as it clearly affected junctional localisation of ZO-1, contributing to the observed induction of barrier dysfunction. These data suggest that junctional ZO-1 is a common downstream signalling target for a number of chemokines and demonstrates their potential importance in disease pathology.

VE-cadherin is also a key mediator of endothelial barrier dysfunction and paracellular leakage (Orsenigo et al., 2012). Its phosphorylation and internalisation lies at the end of many angiogenic, vasogenic and pro-inflammatory signal transduction cascades (Esser et al., 1998). Here, we establish that CCL4 also changes the spatial distribution of VE-cadherin. To the best of our knowledge, this is the first report demonstrating that VE-cadherin is redistributed in response to a CCL chemokine. VE-cadherin redistribution was profound and its manifestations consistent with epitope masking (catenins bind within the C-terminal epitope recognition area) and more importantly internalisation, as also described previously (Dragoni et al., 2021). VE-cadherin internalisation is generally triggered by its phosphorylation and is a pre-requisite of vascular leakage in the periphery (Orsenigo et al., 2012) but also the retinal microvasculature (Dragoni et al., 2021; Ninchoji et al., 2021) in response to VEGF. Consistent with this, phosphorylation of VE-cadherin and loss of barrier integrity has also shown to facilitate increased leukocyte migration via the paracellular route (Wessel et al., 2014; Martinelli et al., 2014; Turowski et al., 2008). Indeed, ICAM-1 crosslinking on BBB ECs, to mimic leukocyte adhesion, resulted in internalisation of VE-cadherin within 5 ​min (Dragoni et al., 2017). In contrast, stabilization of the VE-cadherin/catenin complex has been shown to hinder permeability and leukocyte extravasation (Schulte et al., 2011). CCL4 has been reported to be expressed by BBB endothelial cells in response to ICAM-1 crosslinking, mimicking the molecular effects of lymphocyte migration (Dragoni et al., 2017). The findings in this report have shown that CCL4 has an effect on brain ECs in allowing increased lymphocyte migration, suggesting that there could be an autocrine amplification loop leading to sustained barrier dysfunction.

In this report, we show that VE-cadherin redistribution in hCMEC/D3 cells coincided with enhanced permeability and lymphocyte transmigration induced by CCL4. Overall, this may have wide implications for our understanding of neuroinflammation.

## 5 Conclusion

Throughout our experiments, CCL4 showed consistently strong effects on junctional protein and barrier function demonstrating its capacity to modify CNS vascular permeability and neuroinflammation. Our data is consistent with current literature and highlights a potentially important role for chemokines directly affecting brain vascular endothelial cell function.

The data presented in this paper supports a role of CCL4 in the pathogenesis of neuroinflammatory and neurodegenerative diseases. In relation to neuropsychiatric conditions, it raises the intriguing possibility that CCL4 will contribute to their aetiology. From a clinical point of view, major depressive disorder (MDD) and posttraumatic stress disorder (PTSD) are characterized by raised levels of inflammation, hypothalamic-pituitary-adrenal axis disfunction and reduced neurogenesis. Depressed individuals have shown to have lower levels of CCL4 than healthy individuals (Leighton et al., 2018; Lehto et al., 2010). In fact, one study found lower levels of CCL4 in the CSF of individuals who attempted suicide when compared with health control subjects (Janelidze et al., 2013). A recent meta-analysis showed that patients diagnosed with PTSD have lower levels of CCL4 than healthy controls (Pan et al., 2021). In contrast, CCL4 levels may be increased in other neuro-psychiatric disorders such as schizophrenia (Beumer et al., 2012; Frydecka et al., 2018). Another study focusing on biomarkers to differentiate unipolar and bipolar disorder, found that patients diagnosed with bipolar disorder, had high levels of CCL4 than unipolar counterparts (Poletti et al., 2021). Altogether, there can be a biomarker or therapeutical potential in investigating further the role of abnormal levels of CCL4, its effect on BBB permeability and neurogenesis.

Abnormal levels of chemokines are also found in MS patients. CCL4 has been established as a marker for MS and correlates well with disease duration (Ghafouri-Fard et al., 2021). Pertinent to this body of work, various CCL chemokines have been identified in frontal lobe sections (Subileau et al., 2009), chronic active MS lesions and astrocytes in the CNS of patients with MS (Simpson et al., 2000), suggesting an active role of these chemokines, and by extension of the findings in this report, CCL4, in disease pathogenesis.

CCL4 may also influence neurogenesis; an agonist against CCL4's receptor, CCR5, facilitated neurogenesis in seizure-induced injury in the CNS (Louboutin and Strayer, 2013). These effects may be due to a dampening of the inflammatory processes due to the blocking of CCR5 that, as mentioned above, is known to limit neurogenesis. Of note, a decrease in the expression of this receptor in circulating immune cells reduced BBB leakage and inflammation and promoted neurogenesis rendering the mice less susceptible to seizures (Louboutin et al., 2011).

Altogether, our results suggest a potential for CCL4 as a novel modulating factor in BBB endothelial cells.

## Funding

This work was funded by grants awarded to JG from the 10.13039/100010269Wellcome Trust (090326) and the 10.13039/501100000833Rosetrees Trust/Stoneygate Trust (CM 3001).

## Declaration of competing interest

The authors whose names are listed in the author list certify that they have no affiliations with or involvement in any organization or entity with any financial interest or non-financial interest in the subject matter or materials discussed in this manuscript.
